# Practical tips for teaching ethics and humanism to medical students

**DOI:** 10.12688/mep.19022.1

**Published:** 2022-03-23

**Authors:** Katharine R. Meacham, Ira Sloan, Robyn A. Latessa

**Affiliations:** 1 Mountain Area Health Education Center, UNC School of Medicine, Asheville campus, Asheville, NC, 28803, USA

**Keywords:** clinical ethics, clinical clerkships, medical education, medical ethics, bioethics, narrative ethics, medical school curriculum, humanism in medicine

## Abstract

This article presents the results of a decade’s experiment in creating a longitudinal ethics and humanism curriculum for the core clinical year at UNC School of Medicine, North Carolina, United States. This curriculum applies published research on best practices in medical ethics education. Sample comments from course evaluations of the students who have completed this curriculum provide support for its success at achieving its desired learning outcomes. To create a similar ethics curriculum in the core clinical year at other medical schools, there are twelve practical tips:  preparation: read the research on the ethical challenges for medical students; recruit an interdisciplinary teaching team; create cohorts for this aspect of the curriculum that will stay together for the year; grade only with pass/fail; have the students bring the cases from their clinical experiences; feed them if possible, and structure the time together carefully. Use a narrative ethics methodology and introduce alternative methods for student writing and group process. Connect students with literature in medical humanities and bioethics and encourage publication of their narratives. As with any good creation, the whole is more than the sum of its parts, and each campus can adapt these guidelines for their people and programs.

## Introduction

Medical ethics education used to be taught as discrete courses pre-clinically, using case studies and principles as methodological tools. The 2015 Romanell Report, a comprehensive overview of practices in medical ethics education, argues for a wider set of learning outcomes than the consensus thirty years earlier. It concludes that longitudinal, integrated ethics education is “most likely to result in sustained changes in reasoning and behavior.” If ethics education courses are extended beyond the pre-clinical years, students have the opportunity to identify ethical issues as they arise and to practice the skills previously learned in theory ( Carrese *et al.*, 2015).

The tips presented here have been developed over a decade on one campus. Using the principles of adult education, we have developed narrative-virtue ethics approach. Students write about ethical conundrums and humanistic moments they encounter in clinical settings. These stories get close readings—over a meal—in a group co-created to be safe enough for mutual vulnerability. The year 2020, of course, brought unexpected challenges, so the meals were shared virtually, rather than in person, and the stories, ideas, challenges, and questions were digested together. A year of such meals yields a healthy group, who have learned to share tales, tensions, moral imagination, curiosity, and courage—nourishing each other for the ethical and humanistic practice of medicine. 

## 12 Practical tips

1. **Read** the research on the loss of empathy and ethical sensitivity that often occurs in the first clinical year of medical school ( Holmes *et al.*, 2017; Neumann *et al.*, 2011; Poncelet, 2004).

Neal Chatterjee, citing David Foster Wallace, claims: “The third year of medical school is like being thrown head first into water” ( Treadway & Chatterjee, 2011). That water—which the swimming fish do not see—is the metaphor for “the hidden curriculum” that can erode ethical sensitivity ( Feudtner *et al.*, 1994; Hafferty, 1998). This is the challenge that any ethics curriculum faces.

2. **Recruit** an interdisciplinary teaching team.

The Romanell Report recommends that whatever methods are used to teach medical ethics, the teaching team should be interdisciplinary in order “to reinforce the team approach required in clinical practice” ( Carrese *et al.*, 2015). Others support this best practice—as bioethics has outgrown its early purpose of injecting basic ethical principles into medical practice and has expanded to consider the broader concepts of health and healing, drawing from a broader range of human experience for understanding and perspective ( Dong *et al.*, 2018; Pellegrino, 1999).

Our interdisciplinary team consists of two experienced teachers, a philosopher and a psychiatrist, with a range of experience and expertise across the humanities and medicine as well as improvisational theater. The rules of improv are applicable for both team-teaching and for group-norms: *yes, and…; everything’s a gift…; make the other look good* ( Huffman-Longtin *et al.*, 2018). Those practices—all about collaboration on stage and applicable to collaboration in the clinic as well—help students imagine the perspectives of other stakeholders in a complex situation—developing moral imagination—an essential feature of narrative ethics (Tip 9).

3. **Create** cohorts that will stay together for the entire core clinical year.

This approach can work with students who are in separate rotations throughout the year, or in longitudinal integrated clerkships (LIC). LICs are comprehensive, creative, systemic approaches to innovation in medical education that provide the context and relationships for nurturing the students during the inevitable challenges of the first core clinical year ( Gaufberg *et al.*, 2014; Gheihman *et al.*, 2018; Latessa *et al.*, 2017). Nevertheless, so long as the same group of students can meet for the Ethics and Humanism curriculum—in person or virtually—throughout the core clinical year, the community created will allow for the courage to have shared vulnerability. That is key to its success.

The Ethics and Humanism curriculum receives student affirmation that it maintains and increases ethical sensitivity and nourishes moral imagination in the context of a supportive community. Students get to experience not only their own experience but that of the others in their group. Here is one student analysis:

*In our haste to do and to learn, to adapt and to please, it would be easy to overlook the host of ethical dilemmas before us. The ethics curriculum we experienced reminds us not only to recognize the ethical dilemmas that play out daily in the hospital and the clinic but gives us the time and the tools to analyze these dilemmas. When placed in positions of moral distress, our ethics meetings gave me a safe place to process the tensions I experienced. …and arrive at a host of ethically acceptable outcomes for each situation* (Rebekah Macfie, unpublished work, 2013).

4. **Grade not** and create an environment safe enough for vulnerability.

Medical school can be fertile ground for growing stress, anxiety, moral injury, and cynicism (Tip 1). One of the many causes is the intense competition that medical students experience ( Dyrbye *et al.*, 2005). Assessment experts argue that the appropriate purpose of formative and summative evaluations is to support the learning outcomes ( Maki, 2010). In our Ethics and Humanism curriculum, there are four desired learning outcomes –indicated below, which are evaluated with narrative student comments and interviews every year. For example (student evaluations 2019–20):

- Identify ethical issues in clinical care as they arise, and in reflection:
  ◦ *I think throughout the year I got better at noticing ethical issues that arose. And if I didn’t notice them in the moment, I got better at reflecting on them at the end of the day at home.*
  ◦ *I don’t know if my practice has changed, but I feel like when I have felt uneasy with a situation, I can reflect and unpack why that is. I can share that feeling with people I love and draw conclusions/determine next steps that feel comfortable to me. I think I know how to digest ethical issues better and understand why they feel like issues to me.*

- Engage moral imagination in responding to these issues (e.g., identify at least more than one morally reasonable response-at least in retrospect) and imagine constructive ways of addressing such issues in the future:
  ◦ *I think the narrative model of ethics has really helped with "moral imagination." When you start to consider the viewpoints of all people involved in a situation, it becomes more difficult to simplify problems into a right and wrong answer, and people into being good or bad. I feel like this will continue to be a valuable practice.*
  ◦ *I think just simply reading other people’s writings and their perspective has opened up my imagination a bit. I have been able to reflect on different ways of thinking and observing through my classmates.*

- Practice regular writing, reading, listening, and discussion on the ethical conundrums and humanistic stories that arise in medicine:
  ◦ *I have mostly done reflection through writing before each ethics session. Although sometimes I didn't feel like doing these assignments before I started, I always appreciated having time to reflect after the fact, and I always found that I had something on my mind, even if I had been previously ignoring it, once I started to write. I think I have also been more open to discussing experiences and concerns, both in and out of clinic, with my friends in the program after doing this in ethics sessions.*
  ◦ *I have written a couple of 55-word essays. I like breaking difficult situations/moments/feelings down in that way, and had not done that before. I usually cry when I write them.…I think the writing itself, and the reflection needed in the process, is therapeutic.*

- Co-create with colleagues and facilitators a safe environment in which vulnerability is encouraged.
  ◦ *All of the members of my group were supportive of other students' experiences. I feel like we developed an environment throughout the year where folks felt comfortable sharing the most impactful and hardest moments of this past year. I definitely feel closer to the students in my group now after all the discussions we have had.*
  ◦ *I think it became evident that there was a safe space in my group once I naturally began opening up in my writing. My classmates were super supportive and also were so vulnerable themselves.*

Such evaluative comments, and others that are not as enthusiastic, based on the desired learning outcomes provides feedback for the teaching team on the course, as well as giving an opportunity for the students to do self-evaluation. Students are expected to attend, write, listen, communicate, and participate. In the decade of this program, student participation has been outstanding—without the external motivation of grades.

5. **Provide** an ongoing ‘default’ prompt built on best practices in adult education.

Adult education theory assumes that students are self-directed learners, with internal motivation, who build on prior experience to construct meaning as they seek knowledge. Meaning constructed by adult learners can transform both the learners themselves and the contexts in which they work. Transformation begins with *disorienting dilemmas* and leads to collaborating with others to transform systems ( Mezirow, 2000). Students welcome the opportunity to identify their own disorienting dilemmas and to wrestle in community on how to construct meaning from those experiences.

Our prompts recognize that doing ethics involves using the “logic of emotions” as people identify ethical issues ( Needleman, 1985; Nussbaum, 2001). Our ongoing prompt asks for somatic inklings of emotion as an ethical awakening—e.g., *What is waking you up at night? What is one story from the past month that has grabbed your attention, your heart, your head, your gut? Tell the story and please imagine the perspective of at least one other stakeholder in the story with a perspective different from your own.*

Results from this sort of generic prompt have been rich. For example:

A med student did initial history and physical with an inpatient who had no money, no insurance, no car, and mentioned seven different things wrong with him and with the care he had previously been given. After the attending did his assessment, the student asked if the symptoms the patient had would indicate an aortic dissection. The physician paused; then responded: “Well, I don’t know why this couldn’t be a dissection…You’ve convinced me. I’ll … order a CT angiogram.”

The next morning the attending sought her out: “You were right,” he said. “Unfortunately, aortic dissections can be easy to miss. And this time I would have missed it solely because that patient made me uncomfortable, and I just didn’t want to deal with him, given his mental health and social issues in an ER full of other people.”
  The student concludes:
*This was my first serious “near miss” experience. My emotions were mixed, part excitement … part fright as I realized that our preceptors don’t have all the answers and actually do make mistakes, and a lot of admiration for that physician. Not only did he take a question I asked seriously and stop to consider if he had missed something, but he sat down and talked about why it was near miss and what he can do to prevent it from happening again. I could not have asked for a better learning opportunity.* ( Molly Hamilton, unpublished work, 2019)

6. **Write** additional alternative prompts, to encourage seeing in new ways.

William Carlos Williams, the physician-poet, every day wrote notes to this prompt: “Things I noticed today that I’ve missed until today” ( Coles, 1993). The first prompt of the year for UNC SOM Asheville students is this: *What do you see, hear, smell, touch, and taste in these first few weeks of your clinical clerkship that is new?* Responses include the following:
***Seeing***: *The brutality of surgical entry of the skull in an alive human was a new sight to me. **Hearing**: Hearing the rhythmic nature of the electrical signals of brain cells over a loudspeaker as the electrode pasted by them in the cortex on the way to its target in the thalamus. **Tasting:** the tear I am glad no one else noted slide down the inside of my surgical mask and enter the corner of my mouth when I saw the patient’s eyes when her stimulator was turned on and she was able to raise a cup of water to her mouth without spilling a drop. **Smelling**: burning flesh during laparoscopic surgery* (Thomas Jarrett, unpublished work, 2014).

7. **Feed** them if possible or serve tea. If not possible, feed their hearts—with the arts.

Sharing food literally means creating com-pan-ions (sharing bread together), and it helps create a connection and a context. Meet in a comfortable place—preferably in a home. The American Medical Association cites research on the lack of good eating practices among medical students – especially those in their core clinical year ( Murphy, 2018). A program called PROCOMP uses focus cards with different practices for each month and serves tea ( Seymour *et al.*, 2018). Whether with tea or simple meal, the result is a version of sharing bread together. When students are physically distant, when meeting virtually, as was the case in 2020, the food was artistic, rather than gastronomic: a poem, a song, a work of art can feed the heart and open shared space ( Popp, 2020). Students share their own creations as well as works of art that speak to the ethically challenging and humanistic moments they experience.

8. **Structure** the session around themes that emerge from the writings.

- Students arrive, get food, gather in a circle. Virtually, students arrive and visit.
- A two-minute meditation opens the evening and centers the group.
- One short writing on the theme of the evening opens the student leadership.
- One case-rich with tensions-gets focused narrative ethics analysis.
- Students read aloud the rest of the written contributions, grouped by themes; colleagues reflect with connections, questions, inspirations.
- Conclude with student writing that gives perspective, humor, or hope.

9. **Practice** using *Narrative Ethics* as a methodology and philosophical orientation.

**Figure 1.**
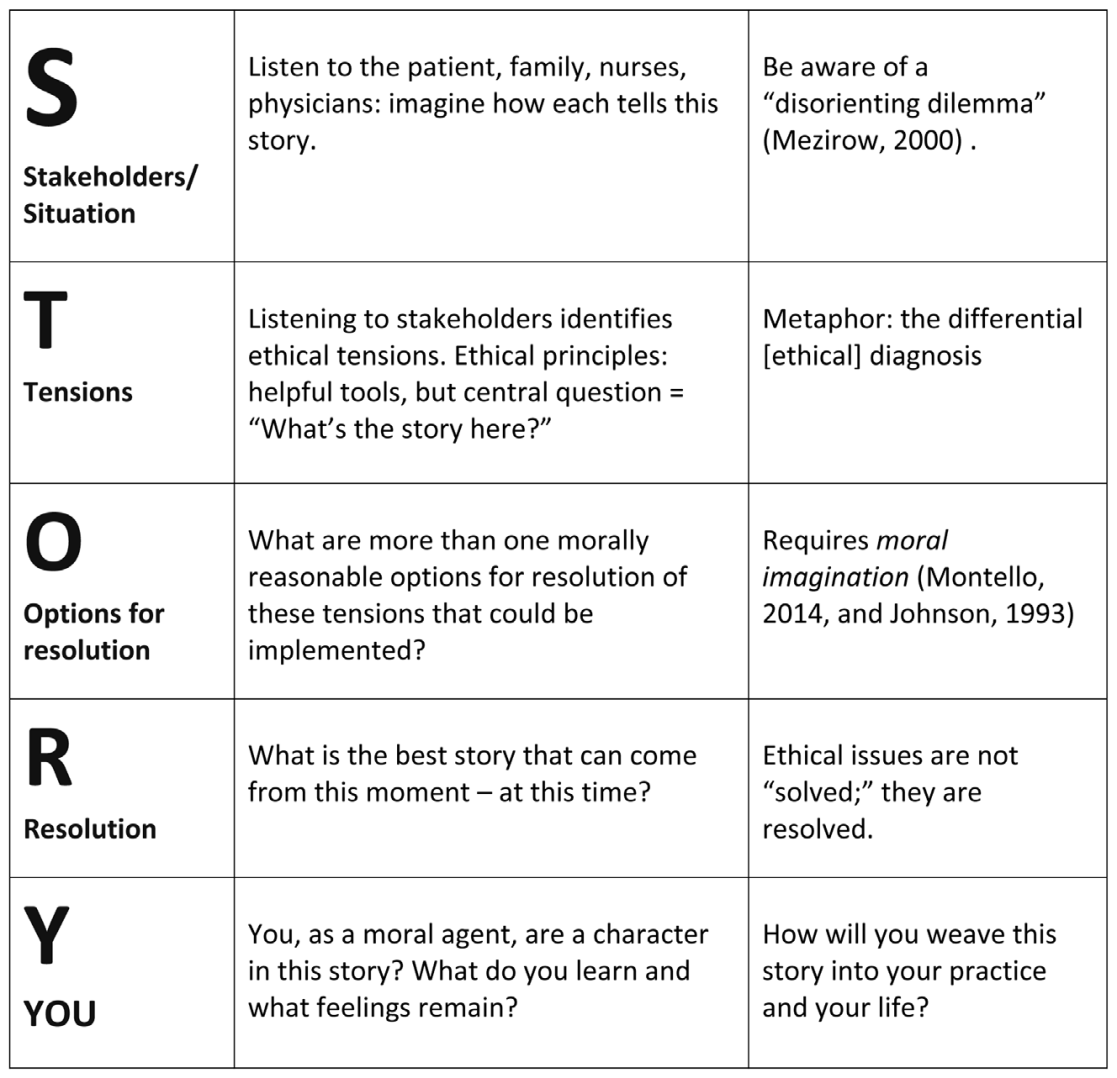
STORY Model for Doing Narrative Bioethics ^1^. This figure represents a process for doing narrative bioethics with clerkship medical students. Begin with a case a student has experienced—rich with tensions. Then work STORY together: S: students identify various stakeholders in this situation. Small groups imagine those different stakeholders’ perspectives, return to the large group and tell the story from that perspective. T: the tensions among the different perspectives are identified as stakeholders speak. O: together, the group imagines options for resolution of these tensions at this time. R: resolution of this situation and reflect on it. Y: return to the first student and see how processing this case can be integrated into future work.

There are many different approaches to practicing *narrative ethics* ( Brody & Clark, 2014). The approach we use is an Aristotelian-informed, modified version of Martha Montello’s methodology—with three facets: creating a *mattering map*, using *narrative competence*, and stimulating *moral imagination* ( Montello, 2014).
Each student begins the year by creating his/ her own *mattering map,* recognizing each brings her own things that matter into the room. This is the basis for establishing a practice of listening for patients’ mattering maps in order to seek understanding. *Narrative competence* is built by paying attention to the characters’ voices, plot, tensions, and the resolutions in each person’s story. *Moral imagination*, building on Mark Johnson’s work, is developed by creating a setting in which students take on the perspectives of each of the stakeholders in a complex narrative we are analyzing, and by encouraging the group to create several different morally reasonable resolutions to the tension-filled situation ( Johnson, 1993). Each narrative ethics case analysis returns to the student: how will the student weave this story into her own story in the future. We have given our way of engaging in this narrative ethics methodology the acronym: STORY ( Figure 1).

10. **Connect** students with research and current issues in bioethics and humanism.

To reinforce the understanding that bioethics is an academic discipline and that humanism in medicine is taken seriously by their professional organizations as well as by academic researchers, each month there are articles sent to the students—geared in two directions: to issues raised by the students and to issues important for the students.

Today’s students are interested in issues beyond the individual clinical physician-patient encounters: they care about the systemic issues, organizational and social ethics, and they are passionate about creating medical care for all people—accessible, just, and affordable. The research and reflections on medical racism—both historically as well as contemporary needs to be presented in small bioethics and humanism groups: in small groups such as these that students are safe enough to be vulnerable about their experiences, questions, and frustrations. For example, one student wrote this 55-word story about her experience with a patient being involuntarily committed:
We told her that the IVC would become official later that afternoon. She responded with many obscenities including a rant somewhat directed toward me about “f**king minorities!” As her prejudice became more overt throughout the week, my ability to empathize waned. She was delusional and severely impaired, but was racism part of her clinical disorder? (Sydney Butler, unpublished work, 2020)

Beyond inviting examination of medical racism, it is important to invite guests with local ties to cultural, racial, and ethnic communities to meet with the students. Share history of the local community, history of medical education, and research on medical racism and the changes coming medical education to address the historical and contemporary challenges. Examples of such can be found from Brown, AAMC and Rutgers.

11. **Use** alternative methodologies as appropriate.

The fifty-five word story works for focusing the reader and the writer ( Fogarty, 2010). For example:
*Two patients. I know them well. I’ve met wives, sisters, sons, and daughters.*
*One’s my favorite, one’s the worst.*
*One imparts life advice. One complains incessantly.*
*One’s laugh reverberates through the halls, the other scowls.*
*Neither went to college. Frank built an electrical engineering empire.*
*Jeff was arrested for public indecency.*
*They are both dying. (John Barber, unpublished work, 2017)*

12. **Encourage publication**

The writing that students produce during their clerkships is often of high quality. In the past decade over twenty students from our program have had their narrative ethics writings presented at conferences and published in journals such as *The New England Journal of Medicine, Academic Medicine, Pulse, Perspectives in Biology and Medicine, Family Medicine, CORE Internal Medicine, the Permanente Journal, and the NYU Langone Online Journal of Medicine, Intima, Annals of Surgery, Psychology Today.*

Two qualitative studies by former medical students and medical education researchers are in progress to learn whether the practices taught in the Ethics and Humanism program persist with graduates of the program (Kelsey Keverline & Jesse Bossingham, unpublished works). These practices might lay a foundation for perspective on their calling into medicine ( Meacham, 2019).

## Conclusion

Clinical year students need practice identifying and responding to ethical issues just as they need practice identifying and responding to the physiological, biological, chemical issues in their patients. Bringing their own cases to a safe place with colleagues and faculty whom they trust provides the setting and the context for accomplishing the goals of improving their identification of ethical issues, practicing moral imagination in responding to ethical issues, and reminding them of the humanistic reasons they entered medicine as a calling in the first place. Using a narrative ethics methodology, led by an interdisciplinary team who practice adult education principles gives respect and allows for vulnerability on the part of the students and the faculty. Evidence from the students bears witness to the success at accomplishing those goals. Witness from the faculty is that this is the most rewarding teaching that we have ever done.

## Data availability

### Underlying data

Dans-Easy: Student Evaluations of SHS4/Ethics and Humanism at UNC SOM Asheville Campus

https://doi.org/10.17615/vc80-q305 ( Meacham *et al.*, 2022)

This project contains the following underlying data:

- Data file 1. (E & H Survey end of year by students 2017-18.docx)
- Data file 2. (E&H End of Yr student evals 2019-20 .docx)
- Data file 3. (Ethics & Humanism end of year student questionnaire 2020-21_1_summary.docx)

Data are available under the terms of the Creative Commons Zero "No rights reserved" data waiver (CC0 1.0 Public domain dedication).

### Extended data

The students were guaranteed confidentiality by the Professors and therefore the full essays have not been provided. Where student names and essay quotes have been included; specific permission was sought.

## Ethics

Permission was obtained from students to include their names and extracts of their essays.

## Footnotes

^1^Developed by Katharine R. Meacham, Ph.D., Ed.M., and used at the UNC School of Medicine Asheville Campus since 2011. See discussion in Tip 9.
