# Supplementary material for: Practical tips for teaching ethics and humanism to medical students
Source: MedEdPublish (2016). 2022 Mar 23;12:23. [Version 1] doi: 10.12688/mep.19022.1 (PMC9370080; doi:10.12688/mep.19022.1)
Supplement: Peer review history

# Peer review: Reviewer response for version 1

**DOI:** 10.21956/mep.20380.r31800

**Published:** 2022-04-04

**Authors:** Margot Hedlin, M.D.

**Affiliations:** 1 Department of Medicine, Memorial Sloan Kettering Cancer Center, New York, NY, USA

This article is an excellent, evidence-based, and highly practical resource for those who want to establish an ethics and humanism program for medical students at their institution. The balance of student writings alongside practical tips was illustrative and well-balanced. Additionally, the citations list provides a rich collection of resources that educators can use as a foundation. I think it is fit for publication as it currently stands, though there are a few questions that came to mind that, if word count allows, may be worth addressing:

1. This paper describes the end result of a 10 year process of growth. Are there mistakes that you learned from along the way? Are there ways that you plan to improve or grow in the future? (tip #4 mentions "...such evaluative comments, and others that are not as enthusiastic", which piqued my curiosity)
2. Sometimes sessions that explore challenging situations and emotions can be triggering for some people - was there a mental health professional you could refer students to if signs arose that a student needed help?
3. Moderation is a skill, and could potentially make or break a group. Are there resources you could point educators to that could help them develop a skillset in moderation?

If evidence from practice is presented, are all the underlying source data available to ensure full reproducibility?

Yes

Is the topic of the practical tips discussed accurately in the context of the current literature

Yes

Are all factual statements correct and adequately supported by citations?

Yes

Are the conclusions drawn balanced and justified on the basis of the presented arguments?

Yes

Are arguments sufficiently supported by evidence from the published literature and/or the authors’ practice?

Yes

Reviewer Expertise:

Medical education, bioethics

I confirm that I have read this submission and believe that I have an appropriate level of expertise to confirm that it is of an acceptable scientific standard.

# Peer review: Reviewer response for version 1

**DOI:** 10.21956/mep.20380.r31796

**Published:** 2022-04-04

**Authors:** Larry R. Churchill

**Affiliations:** 1 Vanderbilt University Medical Center, Nashville, TN, USA

This report from Meacham and colleagues offering “Practical Tips” based on a decade long component in ethics and humanism in the clinical parts of undergraduate medical education is usually helpful. It is grounded in a very sound approach to adult learning, that emphasizes sound gestational, longitudinal principles. The practice of drawing from students’ own ethical awareness and appreciation of what constitutes an ethical problem is preferable to other, more theory- based approaches, or using a stock set of problems as the launching pad for discussion. The emphasis on the virtues needed for ethics and humane approaches to medicine makes a more holistic impression on students than simply emphasizing the “head work” of principled application as an approach to ethical questions. The other tips are altogether essential, such as a Pass/Fail grading system, encouraging individual writing, and creating a safe space that promotes the needed vulnerability for reflection on values. The explication of the uses of narrative methods through the acronym STORY is excellent and original to this essay. I would highly commend this essay to all teachers of medical ethics and humanities as a paradigm of good teaching in clinical contexts.

If evidence from practice is presented, are all the underlying source data available to ensure full reproducibility?

Yes

Is the topic of the practical tips discussed accurately in the context of the current literature

Yes

Are all factual statements correct and adequately supported by citations?

Yes

Are arguments sufficiently supported by evidence from the published literature and/or the authors’ practice?

Yes

Reviewer Expertise:

NA

I confirm that I have read this submission and believe that I have an appropriate level of expertise to confirm that it is of an acceptable scientific standard.
